# Supplementary material for: A retrospective cohort study of prescribing outcomes in outpatients treated with nirmatrelvir–Ritonavir for COVID-19 in an interdisciplinary community clinic
Source: PLoS One. 2023 Oct 19;18(10):e0293302. doi: 10.1371/journal.pone.0293302 (PMC10586632; doi:10.1371/journal.pone.0293302)
Supplement: S1 Table — (DOCX) [file pone.0293302.s001.docx]

**S1 Table. Potential Drug-drug interactions (DDI) with Nirmatrelvir/ritonavir**

| **Severity Level** | **Definition** | **Co-medications** |
| --- | --- | --- |
| 1 | Contraindicated: Use of nirmatrelvir/ritonavir is contraindicated if patient is on this co-medication. Stopping the co-medication is insufficient to mitigate DDI and coadminstration may lead to serious toxicity. | Amiodarone, Apalutamide, Bosentan, Carbamazepine, Clozapine, Dabrafenib, Dronedarone  Enzalutamide, Eslicarbazepine, Fentanyl, Flecainide , Lorlatinib, Lurasidone, Mitotane, Oxcarbazepine, Phenobarbital, Phenytoin, Pimozide, Primidone, Propafenone, Quinidine, Ranolazine, Rifampin, Rifapentine, Sonidegib, St. John's Wort, Tepotinib, Venetoclax |
| 2 | Clinically significant DDI: mitigation strategy is required to minimize clinical consequences (i.e. holding co-medication, dose/interval adjustment, use of alternative agent, management of side effects, additional monitoring) | Abemaciclib, Acalabrutinib, Alfuzosin, Almotriptan  Alprazolam, Amitriptyline, Amlodipine, Amphetamine, Apixaban, Aripiprazole, Atorvastatin  Betamethasone (Topical), Bosutinib, Brexpiprazole, Bromocriptine, Budesonide, Bilastine, Bupropion, Buspirone, Canagliflozin, Cannabis, Ceritinib, Ciclesonide, Cisapride, Clobazam, Clonazepam, Clopidogrel, Clorazepate, Cobimetinib, Combined oral contraceptives, Codeine, Colchicine, Cyclosporine, Dabigatran, Dasatinib, Dexamethasone, Diazepam, Digoxin, Dihydroergotamine Mesylate, Diltiazem,  Dofetilide, Domperidone, Doxazosin, Doxorubicin, Dutasteride, Edoxaban Tosylate, Elagolix, Eletriptan,  Empagliflozin, Encorafenib, Eplenerone, Ergonavine/Methylergonovine, Ergotamine, Escitalopram, Escitalopram, Estradiol, Everolimus,  Felodipine, Fesoterodine, Fluoxetine, Flurazepam  Fluticasone (Inhaled & Nasal), Fostamatinib, Galantamine, Glecaprevir/Pibrentasvir, Hydrocodone, Hydromorphone, Hydroxychloroquine, Hydroxyzine, Ibrutinib, Imatinib, Insulin Degludec, Irbesartan, Irinotecan, Ivermectin, Ketamine, Lercanidipine, Linagliptin, Lomitapide, Loperamide, Lovastatin, Lupron, Meperidine, Metformin, Midazolam, Mirabegron, Mirtazapine, Modafinil, Mometasone, Neratinib, Nifedipine, Nilotinib, Nintedanib, Nitrazepam, Olanzapine, Ondansetron, Oxybutynin, Oxycodone, Palbociclib, Paroxetine, Pimecrolimus, Predisone, Quetiapine, Quinine, Ribociclib, Rifabutin, Risperidone, Rivaroxaban, Rosuvastatin, Rupatadine,  Salmeterol, Semaglutide, Sertraline, Sildenafil, Silodosin, Simvastatin, Sirolimus, Sitagliptin, Sulfasalazine, Tacrolimus, Tadalafil, Tamsulosin, Terazosin, Ticagrelor, Tofactinib, Tramadol, Trazodone, Tresiba, Triazolam, Upadacitinib, Valproate, Valsartan, Vardenafil, Venlafaxine, Verapamil, Vilazodone, Vinblastine, Vincristine, Warfarin, Zanubrutinib, Ziprasidone, Zolpidem, Zopiclone |
| 3 | Minor Drug-Drug Interaction: mitigation strategy not likely required | Anastrozole, Chlordiazepoxide, Chlorpromazine,  Cyclobenzaprine, Donepezil, ,Eltrombopag, Ezetimibe, Indapamide, Letrozole, Losartan, Metoprolol, Mycophenolate, Osimertnib, Paliperidone, Second Generation Antihistamines, Tamoxifen, Vortioxetine |

*Severity Levels, definitions and list of medications are adapted from Ontario Science Table guidelines, Liverpool database, Lexi-interact and Micromedex.(1–4) Recommendations from the Ontario Science Table document and Liverpool databases were prioritized as these are most specific COVID-19 therapeutics and therefore more likely to be comprehensive.(5) Where there was inconsistency between DDI references, the higher severity classification was used to inform the overall severity level.

**References**

1. Ontario COVID-19 Drugs and Biologics Clinical Practice Guidelines Working Group, University of Waterloo School of Pharmacy. Nirmatrelvir/Ritonavir (Paxlovid): What Prescribers and Pharmacists Need to Know [Internet]. Ontario COVID-19 Science Advisory Table; 2022 Feb [cited 2022 Dec 20]. Available from: https://covid19-sciencetable.ca/sciencebrief/nirmatrelvir-ritonavir-paxlovid-what-prescribers-and-pharmacists-need-to-know-2-0

2. Liverpool Drug Interactions Group. COVID-19 Drug Interactions [Internet]. University of Liverpool; Available from: https://www.covid19-druginteractions.org/checker

3. Lexi-Interact [Internet]. Lexi-Comp, Inc.; Available from: https://www-e-therapeutics-ca.myaccess.library.utoronto.ca/NEW/TOOL/DI

4. Drug Interactions [Internet]. Merative MicroMedex; Available from: https://www.micromedexsolutions.com/micromedex2/librarian/CS/30A468/ND_PR/evidencexpert/ND_P/evidencexpert/DUPLICATIONSHIELDSYNC/33E68F/ND_PG/evidencexpert/ND_B/evidencexpert/ND_AppProduct/evidencexpert/ND_T/evidencexpert/PFActionId/evidencexpert.FindDrugInteractions?navitem=topInteractions&isToolPage=true

5. Sheehan N, Tseng A, Hewlett K, Dumas M, Harvey B, Godin M, et al. Drug-Drug Interactions in Hospitalized COVID-19 Patients Receiving Investigational Drugs (CATCO-DDI). In Virtual Meeting; 2021.
