# Supplementary material for: A retrospective cohort study of prescribing outcomes in outpatients treated with nirmatrelvir–Ritonavir for COVID-19 in an interdisciplinary community clinic
Source: PLoS One. 2023 Oct 19;18(10):e0293302. doi: 10.1371/journal.pone.0293302 (PMC10586632; doi:10.1371/journal.pone.0293302)
Supplement: S2 Table — (DOCX) [file pone.0293302.s002.docx]

**S2 Table. Prevalence of DDIs**

| **Prevalence of Interacting Comedications % (n)**  **N=840 total DDI** | | |
| --- | --- | --- |
| Cardiovascular drugs, 54.8% (460) | Lipid modifying agents (statins), 29.3% (246) | Rosuvastatin (144), Atorvastatin (93), Simvastatin (9) |
|  | Calcium Channel Blockers, 13.9% (117) | Amlodipine (99), Diltiazem (14), Nifedipine (3), Verapamil (1) |
|  | Alpha-blockers, 5.7% (48) | Tamsulosin (30), Terazosin (7), Silodosin (6),  Afluzosin (3), Doxazosin (2) |
|  | Phosphodiesterase Type 5 Inhibitors, 2.0% (17) | Tadalafil (9), Sildenafil (8) |
|  | Other Cardiovascular agents , 3.8% (32) | Indapamide (12), Valsartan (7), Ezetimibe (3), Irbesartan (3), Eplenerone (2), Losartan (2), Metoprolol (2), Lercanidipine (1) |
| Central Nervous System Drugs, 18.5% (155) | Benzodiazepine receptor agonists 5.8% (49) | Zopiclone (25), Clonazepam (17), Alprazolam (2), Clobazam (2), Chlordiazepoxide (1), Triazolam (1), Zolpidem 1 |
|  | Antidepressants, 4.4% (37) | Trazodone (10), Bupropion (7), Mirtazapine (5), Venlafaxine (4), Amitriptyline (3), Escitalopram (2), Fluoxetine (2), Buspirone (1), Paroxetine (1), Sertraline (1), Vilazodone (1) |
|  | Opioids 3.3% (28) | Oxycodone (13), Codeine (7), Hydrocodone (3), Hydromorphone (2), Tramadol (3) |
|  | Antipsychotics, 1.7% (14) | Aripiprazole (4), Quetiapine (4), Brexpiprazole (2), Chlorpromazine (1), Olanzapine (1), Risperidone (1), Paliperidone (1) |
|  | Other CNS agents, 3.2% (27) | Cannabis (17), Modafinil (3), Amphetamine (3), Ketamine (1), Almotriptan (1), Eletriptan (1), Valproate (1) |
| Oral Antithrombotic Agents, 5.1% (43) |  | Apixaban (21), Clopidogrel (9), Warfarin (7),  Rivaroxaban (4), Edoxaban (2) |
| Corticosteroids, 4.4% (37) |  | Budesonide (14), Fluticasone (11), Predisone (6), Betamethasone (2), Ciclesonide (1), Dexamethasone (1), Mometasone (2) |
| GI/GU agents, 2.9% (24) |  | Mirabegron (10), Dutasteride (5), Domperidone (3), Loperamide (2), Fesoterodine (2), Oxybutynin (1), Ondansetron (1) |
| Immunosuppressants, 2.9% (24) |  | Tacrolimus (6), Hydroxychloroquine (5), Tofactinib (4), Sulfasalazine (4), Upadacitinib (2), Mycophenolate (1), Pimecrolimus (1),  Cyclosporine (1) |
| Antihistamines 2.5% (21) |  | Second generation antihistamines (10),  Bilastine (6), Hydroxyzine (2), Rupatadine (3) |
| Hormones 2.4% (20) |  | Combined oral contraceptives (14), Estradiol (2), Anastrozole (1), Letrozole (1), Lupron (1),  Tamoxifen (1) |
| Autonomic agents, 2.5% (21) |  | Salmeterol (14), Donepezil (3), Cyclobenzaprine (2), Galantamine (2) |
| Antineoplastic agents, 2.0% (17) |  | Ibrutinib (2), Nilotinib (2), Ribociclib (2), Zanubrutinib (2), Acalabrutinib (1), Dasatinib (1), Doxorubicin (1), Imatinib (1), Lorlatinib (1), Olaparib (1), Osimertnib (1), Palbociclib (1), Nintedanib (1) |
| Other 2.1% (18) |  | Colchicine (5), Metformin (3), Sitagliptin (2),  Tresiba (1), Canagliflozin, (1), Empagliflozin (1), Semaglutide (1), Bromocriptine (1), Ivermectin (1), Quinine (1), Eltrombopag (1) |
