# Supplementary material for: A retrospective cohort study of prescribing outcomes in outpatients treated with nirmatrelvir–Ritonavir for COVID-19 in an interdisciplinary community clinic
Source: PLoS One. 2023 Oct 19;18(10):e0293302. doi: 10.1371/journal.pone.0293302 (PMC10586632; doi:10.1371/journal.pone.0293302)
Supplement: S3 Table — (DOCX) [file pone.0293302.s003.docx]

**S3 Table. Characteristics of Patients Hospitalized for COVID-19**

| **Patient** | **Baseline Characteristics^1^** | **Time from symptom onset to start of therapy (days)** | **Nirmatrelvir/ritonavir Treatment** | **Reason for Admission** |
| --- | --- | --- | --- | --- |
| 1 | 72-year-old with 2 risk factors, 2 vaccine doses | 3 | Discontinued therapy after 2 days | ADE – gastrointestinal |
| 2 | 93-year-old with 3 risk factors, 4 vaccine doses | 3 | Completed 5-day course. | Progression of COVID-19 symptoms. |
| 3 | 94-year-old with 3 risk factors, 2 vaccine doses | 1 | Completed 5-day course. | Progression of COVID-19 symptoms. |
| 4 | 82-year-old with 2 risk factors, 4 vaccine doses | 1 | Completed 5-day course. | Progression of COVID-19 symptoms. |
| 5 | 75-year-old with 2 risk factors, 4 vaccine doses | 3 | Discontinued after 3 days. | ADE – gastrointestinal |
| 6 | 47-year-old with 2 risk factors, no vaccine doses | 0 | Discontinued after 1 dose. | Progression of COVID-19 symptoms |
| 7 | 72-year-old with 2 risk factors, 4 vaccine doses | 5 | Completed 5-day course. | Progression of COVID-19 symptoms |

^1^No patients that were hospitalized were immunocompromised

ADE = adverse drug reaction
